# Ecological Factors at a Fine Spatial Scale Influencing Leopard (*Panthera pardus*) Habitat Use in the Bardia–Banke Complex, Nepal

**DOI:** 10.1002/ece3.73285

**Published:** 2026-03-18

**Authors:** Rabin Bahadur K. C., Jyoti Sharma, Bed Kumar Dhakal, Rabin Kadariya, Ajit Tumbahangphe, Shyam Kumar Thapa, Naresh Subedi

**Affiliations:** ^1^ National Trust for Nature Conservation Lalitpur Nepal; ^2^ Faculty of Applied Ecology, Agricultural Sciences and Biotechnology University of Inland Norway Norway; ^3^ Department of National Parks and Wildlife Conservation Kathmandu Nepal; ^4^ Tribhuvan University Kathmandu Nepal; ^5^ Zoological Society of London Nepal

**Keywords:** camera trap, detection probability, habitat use, leopard, sympatric carnivores

## Abstract

Conservation of large carnivores like leopard requires the clear knowledge of the spatial ecology of leopards ( *Panthera pardus* ), especially in larger and interconnected habitats to inform effective conservation planning. Here, we assessed leopard habitat use across Bardia–Banke Complex (BBC), Nepal, using single‐season occupancy models from 405 camera trap stations deployed over 7423 trap nights. We conducted the camera trap survey from December 2020 to June 2021 in 4 sq.km grids. We prepared the detection history (detection/non‐detection) record of leopard and then evaluated the impact of the selected 10 plausible ecological and anthropogenic covariates on leopard occupancy. Our analysis revealed that prey availability, distance to human settlements, and rivers were the key determinants of leopard's habitat use. The model‐averaged leopard occupancy was estimated at 0.55 (SE ± 0.134), with higher use in prey‐rich zones and forest fringes, and distance from the riparian area suggesting spatial displacement from core habitats. Detection probability varied significantly by camera model, with Browning cameras outperforming others. These findings highlight the leopard's ecological plasticity and its persistence in human‐modified landscapes, underscoring the need for an integrated conservation approach that maintains prey base, secures ecological corridors, and addresses conflict mitigation around the parks.

## Graphical abstract

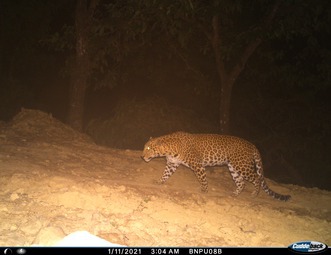

## 1 Introduction

**FIGURE 1:**
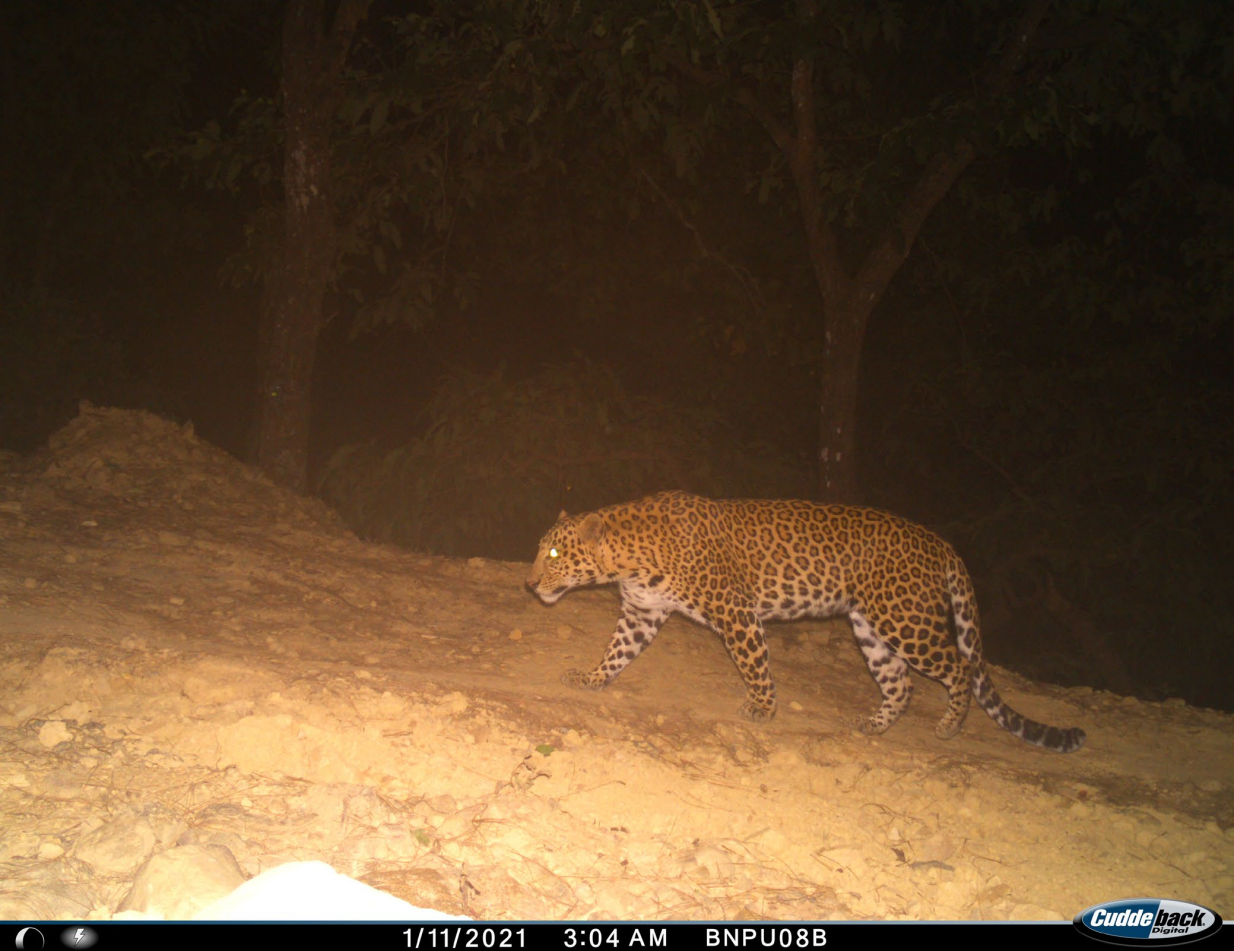
Common leopard (Panthera pardus) photographed in camera traps in Bardia National Park, Nepal.

The common leopard (*Panthera pardus*, called “leopard” hereafter, Figure 1) is a habitat generalist and the widely distributed felid (Sunquist and Sunquist 2002; Stein and Hayssen 2013). Leopards can survive in extreme climatic conditions and habitat types because of their diet flexibility (Hayward et al. 2006) and its ability to live in environments ranging from rainforest to deserts, and even in areas of high human impact (Athreya et al. 2013; Gubbi et al. 2020). Because of their high adaptability, leopards are distributed throughout Asia and Africa in various habitat types (Sanquist and Sanquist 2002; Jacobson et al. 2016). However, because of constant pressure, the survival of the leopard is guarded by threats like habitat loss, poaching, human wildlife conflict, depletion of prey, roadkill, and trophy hunting (Walston et al. 2010; Athreya et al. 2011; Jacobson et al. 2016; Lamichhane et al. 2021). The major threat to this species is directly caused by anthropogenic activities (Ray et al. 2005; Gubbi et al. 2014; Stein et al. 2024). The global range of leopards has contracted by over 30% in the past three generations (22.3 years) (Stein et al. 2024). Leopards with the decreasing population are listed as “Vulnerable” in the IUCN Redlist (Stein et al. 2024). Although protected areas (PAs) are crucial for many large carnivores to sustain stronghold populations, it does not seem to be valid for leopards, because conservation of leopards also depends on measures outside PAs (Woodroffe and Ginsberg 1998; Balme et al. 2010; Swanepoel et al. 2013; Strampelli 2015). Because of large movement territory, low population densities, high energy needs, and interference competition, conserving wide‐ranging leopards within protected boundaries is challenging, which frequently causes them to move beyond protected borders (McCarthy et al. 2005; Jackson and Lama 2016; Sigdel et al. 2025).

In several PAs in Asia, leopards compete with tigers ( *Panthera tigris tigris* ) (Barber‐Meyer et al. 2013; Stein et al. 2024), and in Nepal, they share the habitat with the tigers in 5 protected areas in lowlands (Terai) of Nepal (Lamichhane et al. 2021; DNPWC and DFSC 2022). This interference competition with tigers may drive leopards toward human‐dominated landscapes, potentially increasing livestock depredation (Franchini and Guerisoli 2023). Despite their high tolerance to human‐modified environments, conflict incidents are trending high in Nepal and making them more vulnerable. In Nepal, leopards occur across much of the country, ranging from the lowlands of Terai to the mountains up to the elevations of approximately 4260 m, with an estimated population of around 1000 mature individuals (Jnawali et al. 2011; Chetri et al. 2023). In Nepal, more than 43% of the habitat lies in non‐protected forest and agricultural areas (Baral et al. 2023), and 6052 km^2^ area of leopards falls outside the protected areas (Malla et al. 2023). Within the Chure range, where the leopard shares the habitat with tigers, the detection probability was higher outside the PA (Lamichhane et al. 2021). Therefore, to meet their dietary needs, leopards frequently kill livestock (Khorozyan et al. 2015) and occasionally attack humans. Such conflict occasionally results in retaliatory killings (Thapa 2014).

According to National Tiger Survey—2022, Nepal harbors 355 tigers, and the two national parks in the western Terai Arc Landscape (TAL) of Nepal, that is, Bardia and Banke National Parks, hereinafter termed as Bardia—Banke Complex (BBC) holds the second largest tiger population in Nepal (*n* = 150) (DNPWC and DFSC 2022). Within the BBC, a total of 388.16 km^2^ area has been identified as the overlapping habitat between the tiger and leopard (Kandel et al. 2025), suggesting high competition between leopards and tigers. Bardia National Park hold 125 tigers with a density of 7.15 tigers per 100 sq.km (SD = 0.38) and Banke National Park support 25 tigers with a density of 0.97 tigers per 100 sq.km (SD = 0.12) (DNPWC and DFSC 2022); therefore, the impact of tigers on sympatric leopards needs to be assessed. As tigers and leopards are the two sympatric felids in Asia, sharing a similar habitat and diet, which ultimately leads to high competition in conditions of limited resources (Wegge et al. 2009; Odden et al. 2010; Lovari et al. 2015; Goodrich et al. 2022; Stein et al. 2024). Leopard distribution is shaped primarily by interference competition with tigers, resulting in spatial exclusion from core tiger habitats (Odden et al. 2010; Kafley et al. 2019; Lamichhane et al. 2021). Previous studies have provided valuable insights into the broad‐scale distribution and ecological drivers of leopards across Nepal (Lamichhane et al. 2021). However, finer resolution assessments within specific landscapes (BBC) are still needed to understand how environmental and anthropogenic variable's structure habitat use at spatial grains relevant to individual space use. Within the BBC, the significant increase in livestock attacks has been observed over the year (Dhakal et al. 2023; Chaudhary et al. 2025). The association of tiger and leopard have been extensively studied in other ranging areas such as Nagarhole National Park, Rajaji National Park, and Chitwan National, but in other overlapping range areas, the study has been neglected. The global TX2 initiative, ambitious goal, set in 2010 by 13 tiger‐range countries to double the number of wild tigers by 2022, led to extensive habitat management and intensified protection measures across the landscape. As the 2022 population estimates now confirm a significant upward trend in tiger numbers, there is urgent need to assess the resulting impact of these increased densities on sympatric leopards. In this context, our study conducted in 2020–2021 provides a critical baseline of leopard occupancy during this period of transition. Therefore, we use an occupancy modeling framework to bridge the knowledge gap on the influence of ecological and anthropogenic factors shaping the leopard occupancy. We hypothesize that the area with tiger relative activity index impact the occupancy of the leopard negatively and leopard tend to live nearby the human settlement. Our objectives are to (a) develop the occupancy maps of leopard distribution in BBC and (b) assess the impact of environmental and anthropogenic variables on the leopard occupancy.

### 1.1 Study Area and Methodology

Bardia—Banke Complex (BBC) comprises two national parks of the western region of Nepal, i.e., Bardia National Park (BNP) and Banke National Park (BaNP). The study area is administratively located in the country's Lumbini Province, covering 1518 sq.km (BaNP 2022; BNP 2022). BBC is a part of western TAL of Nepal; BNP is connected to Katarniaghat Wildlife Sanctuary (KWS), India, via Khata corridor, and BaNP is connected to Suhelwa Wildlife Sanctuary, India, via Kamdi corridor (Thapa and Tuladhar 2021; Bhatt et al. 2023).

The BBC is one of the 42 source sites for tiger recovery globally (Walston et al. 2010) and is identified as a Level 1 tiger conservation unit (Wikramanayake et al. 1998). The BBC comprises subtropical vegetation to temperate climate and dominant vegetation types, within an elevation ranging from 100 m in Terai to 1500 m in the Chure hills (Shah et al. 2024). Within the BBC, sal forest is the dominant vegetation type, including riverine forests, riverine floodplain, interspersed grassland, and mixed hardwood forest. The study area is considered a biodiversity hotspot that harbors more than 60 species of mammals, 513 species of birds, and 42 species of herpetofauna (BaNP 2022; BNP 2022).

The BBC consists of predators like the tiger, leopard, striped hyena ( *Hyaena hyaena* ). Prey species like spotted deer ( *Axis axis* ), hog deer ( *Axis porcinus* ), wild boar ( *Sus scrofa* ), barking deer (*Muntiacus muntjac*), four‐horned antelope ( *Tetracerus quadricornis* ), and swamp deer ( *Rucervus duvaucelii* ) are found in this area (Wegge et al. 2009). Other species such as dhole, golden jackal ( *Canis aureus* ), fishing cat ( *Prionailurus viverrinus* ), sloth bear ( *Melursus ursinus* ), and Himalayan black bear ( *Ursus thibetanus laniger* ) (Yadav et al. 2017, 2018, 2019; Shah et al. 2025a, 2025b) are associated carnivores in the park. Mega herbivores like the greater one‐horned rhinoceros ( *Rhinoceros unicornis* ) and the Asian elephant ( *Elephas maximus* ) are also present in the area.

### 1.2 Field Survey and Data Collection

**FIGURE 2:**
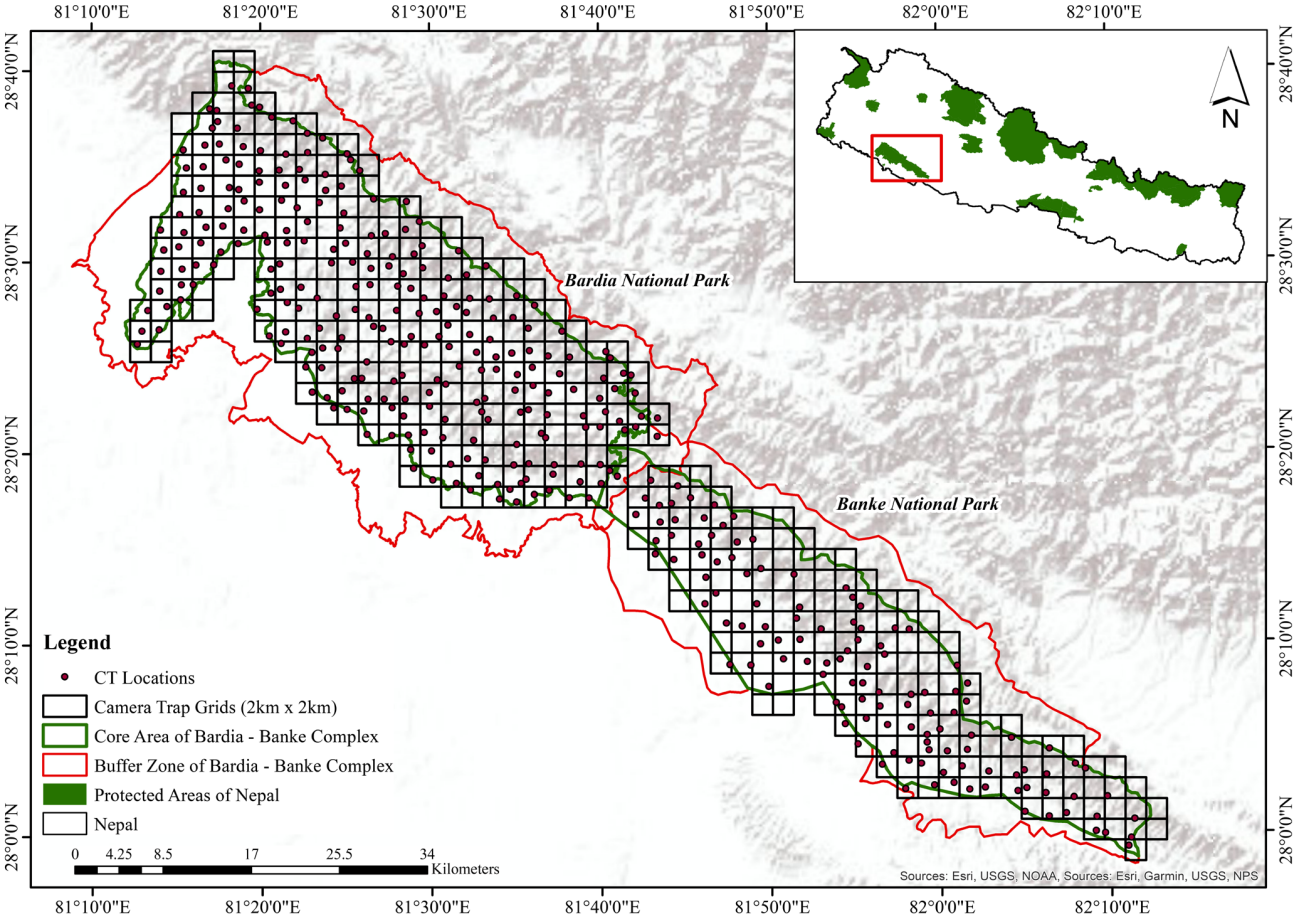
Map showing Bardia—Banke Complex, including Bardia National Park in the western section and Banke National Park in the eastern section. The dots are the camera trap locations within 2 km × 2 km grids. The inset map shows the protected areas of Nepal.

To monitor the tiger, a camera trapping survey was conducted in the winter season from December 2020 to June 2021, following the tiger and prey‐based monitoring protocol issued by the Government of Nepal (DNPWC 2017; NTNC 2022). To set up the camera traps, we divided the whole study area (BBC) into 2 km × 2 km grids and deployed a pair of camera traps at 405 locations across the core area of the BBC (Figure 2). Because of the fixed available number of camera traps for the survey, we divided the 405 grids into five blocks (Block 1 = 53, Block 2 = 49, Block 3 = 43, Block 4 = 31, Block 5 = 60, Block = 26, Block 7 = 67, Block 8 = 76) and surveyed subsequently (NTNC 2022). As the survey was focused on tigers, selectively camera traps were placed on forest fire lines, forest roads, dry riverbeds, and animals' trails, and nearby water sources to potentially increase the detectability of tigers (Karanth et al. 2011). In each grid, a pair of camera traps was deployed 4–5 m apart at a height of 40–60 cm above the ground (Evans, Mosby and Mortelliti, 2019) to capture both flanks of the passing tigers. Camera models like Browning (*n* = 64), Cuddeback (*n* = 222), Reconyx (*n* = 10) and Panthera (*n* = 81) were used and kept active for 24 h throughout the sampling period. Camera traps were set up to take 3 pictures per trigger, with no delay (FAP mode), and deployed for 15–22 nights in each of the grid cells (DNPWC 2017; NTNC 2022). The 4 sq.km grid size is substantially smaller than leopard home ranges in the Terai Arc Landscape; occupancy estimates reflect fine‐resolution, within home range habitat use patterns rather than broad landscape‐level distribution.

### 1.3 Selection of Covariates

We selected a mix of 11 plausible covariates reflecting the characteristics of the landscape, habitat conditions, and persistent anthropogenic pressures. We applied the z‐transformation to the selected continuous covariates to normalize for consistency and comparability (Table 1) (Sunarto et al. 2012). We assessed the collinearity of the covariates before running occupancy models (Dormann et al. 2013). Elevation and Terrain Ruggedness Index (TRI) were highly correlated, so we discarded the elevation in the model‐building procedure (used 10 covariates) (Pearson's |r| = > 0.7) (Schober and Schwarte 2018).

**TABLE 1:** Covariate selection with a priori hypotheses. List of covariates used in the analysis for the single season single species occupancy of leopard in Bardia—Banke Complex.

Types	Covariates	Rationale	Submodel	Source details
Categorical	Camera models	Different camera models influence detection probability and data quality. Reliable camera models increase detection rates and reduce bias in occupancy estimates, crucial for robust analysis (Rovero et al. 2013; Tan et al. 2022).	*p*	Field data
	Habitat types (flat, hilly, streambed)	Leopards use diverse habitats including forests, agricultural landscapes, and human settlements. Habitat type affects prey availability, cover and human disturbance, all influencing occupancy (Bista et al. 2022).	*ψ*	Field data
Continuous	Elevation	Study shows higher occupancy at different elevations depending on human disturbance and prey availability; higher elevations may offer refuge from human pressure (Greenspan et al. 2023).	*ψ*	Digital Elevation Model (DEM) downloaded from worldclim (30 s i.e., approx. 1 km resolution): https://www.worldclim.org/data/worldclim21.html. Using ArcGIS 10.5, we extracted the value of the point (camera trap location) from DEM file.
	Terrain ruggedness index (TRI)	TRI represents topographical heterogeneity, affecting leopard movement, prey vulnerability, and cover availability during hunting. Rugged terrain often positively correlated with leopard occupancy by providing refuge and hunting advantages (Hinde et al. 2023).	ψ	Extracted from Digital Elevation Model (DEM) downloaded from worldclim: https://www.worldclim.org/data/worldclim21.html. Using ArcGIS 10.5, we extracted the value of the point (camera trap location) from TRI raster layer.
	Distance to road	Proximity to roads influences human disturbance and mortality risks. Increased distance to roads generally favors leopard occupancy because of less disturbance and mortality risk from vehicles or poaching (Thapa et al. 2021).	*ψ*	Downloaded the road layer from the Humanitarian Data exchange: https://data.humdata.org/dataset/hotosm_npl_roads. Using the Spatial Analyst Tool (near tool) extension of ArcGIS v.10.5, we measured the distance to nearest road from our camera trap locations.
	Distance to settlement	Distance to settlement affects disturbance and risk of conflict. Leopards may avoid or adapt to human pressure; occupancy is often lower closer to settlements unless prey availability (Joshi et al. 2025).	*ψ*	Downloaded the road layer from the Humanitarian Data exchange: https://data.humdata.org/dataset/settlements‐in‐nepal. Using the Spatial Analyst Tool (near tool) extension of ArcGIS v.10.5, we measured the distance to nearest human settlement from our camera trap locations.
	Distance to river	River provides cover, water, prey concentration, and travel corridors. Leopards may show higher occupancy near rivers, although effects can vary on the basis of human activity (Bista et al. 2022; Joshi et al. 2025).	*ψ*	Downloaded the river layer from the Humanitarian Data exchange: https://data.humdata.org/dataset/nepal‐watercourses‐rivers. Using the Spatial Analyst Tool (near tool) extension of ArcGIS v.10.5, we measured the distance to nearest river from our camera trap locations.
	Distance to waterholes (waterholes: the artificial ponds constructed by park authority to increase the water sources for prey base)	Waterholes attract prey species and are important for leopard hunting and hydration, positively influencing occupancy (Joshi et al. 2025).	*ψ*	The shapefile (point) of waterholes was obtained from National Trust for Nature Conservation (NTNC) and using the Spatial Analyst Tool (near tool) extension of ArcGIS v.10.5, we measured the distance to nearest waterholes from our camera trap locations.
	Normalized difference vegetation index (NDVI)	NDVI is a proxy for vegetation productivity, linked to prey abundance and cover. Relationship with leopard occupancy can be positive or complex depending on human disturbance and habitat quality (Greenspan et al. 2023; Hinde et al. 2023).	*ψ*	Downloaded from MODIS: https://modis.gsfc.nasa.gov/data/dataprod/mod13.php. Using ArcGIS 10.5, we extracted the value of the point (camera trap location) from NDVI raster layer.
	Prey index	Prey abundance is a crucial driver of leopard occupancy. Areas with higher prey availability support higher leopard occupancy because of better food resources (Lamichhane et al. 2021).	*ψ*	Camera trap survey. Firstly, we calculated the capture rate of all prey species by calculating the number of photos event per 100 trap nights for each prey species (spotted deer, barking deer, swamp deer, wild boar, four‐horned antelope, sambar deer, and hog deer). The photos of a species taken within 30 min at a camera location were considered a single photo event (Carter et al. 2012). Later the photo events of all prey species were summed for each location to obtain the prey index (Shah et al. 2024).
	Population density	Human population density negatively influenced leopard occupancy by increasing disturbance, habitat loss, and conflict potential. Some leopards adapt to peri‐urban areas though with varied success (Bista et al. 2022; Hinde et al. 2023; Joshi et al. 2025).	*ψ*	Downloaded the population density layer of Nepal 2020 population census from the Humanitarian Data Exchange: https://data.humdata.org/dataset/worldpop‐population‐density‐for‐nepal
	Tiger index	Previously, multiple studies provided strong evidence for the relationship of leopard and tiger. (Joshi et al. 2025) found leopard detection was negatively correlated with tiger presence. Similarly, (Thapa et al. 2021) suggested that the leopard occupancy declined in sites with tigers.	*ψ*	Camera trap survey. The tiger index was calculated using the same methodology as that applied for the prey index (Carter et al. 2012; Shah et al. 2024).
	Occasion	Accounts for temporal variation in detection across survey intervals.	*p*	Created from survey matrix (1–25).

### 1.4 Data Analysis

We applied a single species single season occupancy model to assess leopard habitat use, as it implies presence‐absence data for robust estimates of the probability of use and also accounts for the imperfect detection (Mackenzie et al. 2002; Mackenzie 2006). We modeled leopard habitat use using a maximum likelihood approach in the statistical software, *R* Version: 2025.09.2 + 418 (R Core Team 2025), using the extension package “unmarked” (Fiske and Chandler 2011; Kellner et al. 2023). As each grid contained one camera trap station, having a pair of camera traps, therefore, we combined the detection from the pair camera traps into a single day detection record for that grid. For each 24 h period, we coded detection of leopard as 1 if at least one of the camera traps recorded the species in that grid, and 0 if neither camera trap recorded (Kalle et al. 2014; Joshi et al. 2025). Similarly, days when the camera traps were not functioning were treated as missing data and excluded from that grid's detection history (Shah et al. 2025a, 2025b). To construct capture histories for occupancy analysis, every five consecutive survey days were pooled into a single trapping Occasion. Because of staggered block design, cameras were active in each grid for 15–22 trap nights, resulting in 3–5 active Occasions Occasion per grid within 25‐Occasion study framework. A detection (1) was recorded for a trapping Occasion if a leopard was detected at least once during the five‐day period, and non‐detection (0) otherwise. Occasions during which cameras were not deployed were treated as missing values (NA) in the detection history. Pooling days in this manner was used to ensure that the capture probability exceeded 0.10 per trapping occasion, following the recommendations of Otis et al. (1978) and White et al. (1982).

To estimate the detection and occupancy probability (habitat use), a two‐step model‐fitting approach was adapted, following Mackenzie et al. (2002) and Sunarto et al. (2012). In the first step, we prepared the model for detection probability by keeping constant occupancy [*Ψ* (.), *p* (covariates)] (Hines et al. 2010). The top detection model served as the basis for subsequent models that incorporated covariates to explain leopard habitat use. Therefore, in the second step, we constructed the occupancy model by keeping the most influential covariates from the detection probability (Karanth et al. 2011; Srivathsa et al. 2018). We fitted a total of 21 (3 detection and 18 occupancy) constructive models to assess the leopard's detection and occupancy estimation. All prepared model comparisons were based on Akaike Information Criterion (AIC) values (ΔAICc < 2) (Burnham and Anderson 2002). We also checked the c‐hat of the top model to test the goodness of fit (MacKenzie and Bailey 2004; Crujeiras et al. 2010). For a model with an adequate description of data, c‐hat is expected to be close to 1, whereas greater or smaller values represent the overdispersion and underdispersion respectively (Burnham and Anderson 2002). We used the *β* coefficients of the model containing the particular covariate to assess the relative influence of covariates on occurrence.

## 2 Results

Within 405 camera trap stations, a total of 7423 camera trap nights were conducted and detected the leopard from 82 stations with a naïve occupancy of 0.20.

### 2.1 Probability of Detection

**FIGURE 3:**
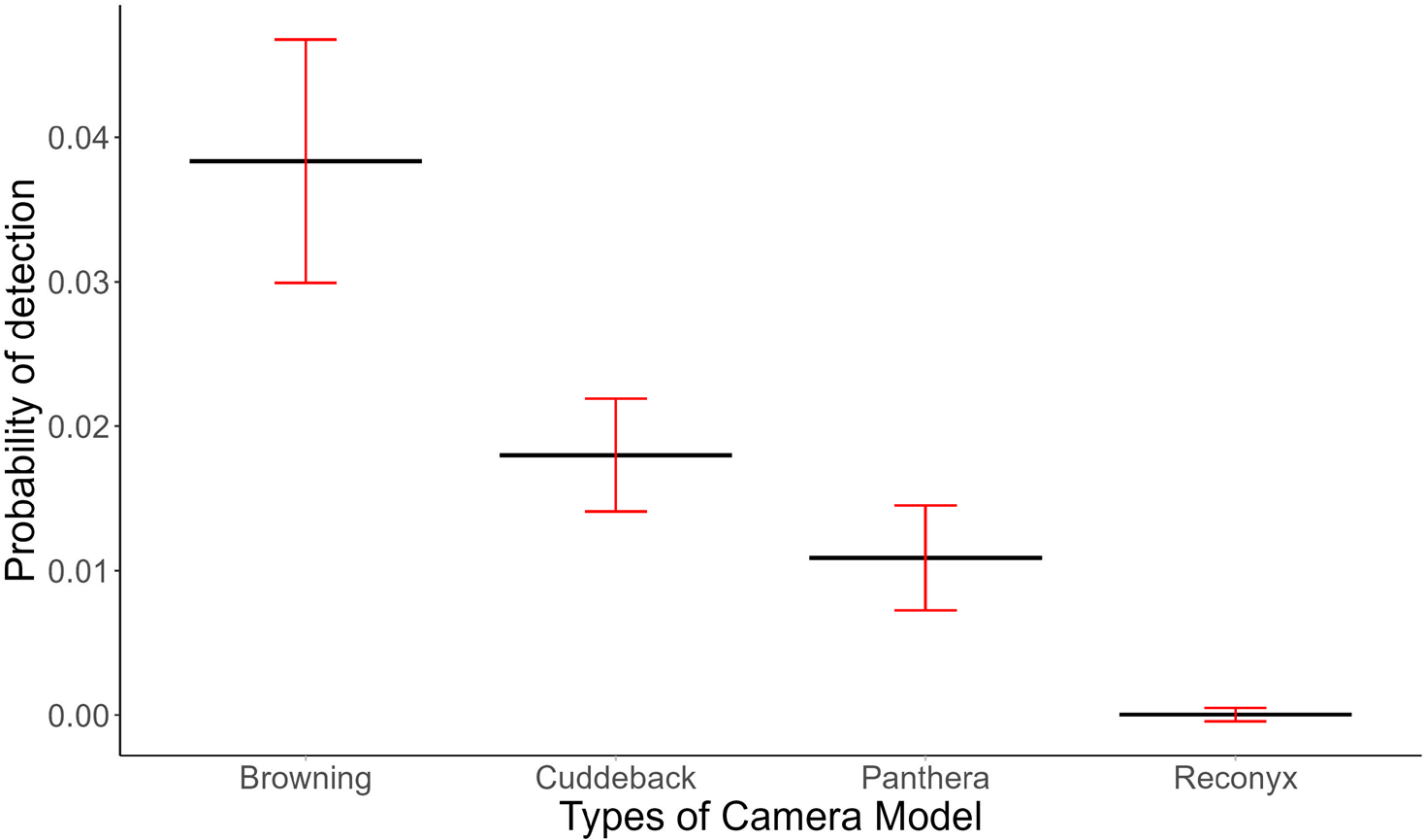
Relationship between types of camera model and probability of detection in BBC.

We modeled the factors probably influencing the detection probability by assuming the occupancy constant. The top model for probability of detection (*Ψ* (.), *P* (covariates)) showed the effects of camera models influenced the leopard detection (Table 2). The Browning camera traps (*β* = −3.23, SE ± 0.25) performed better compared to Cuddeback (*β* = −0.78, SE ± 0.24), Panthera (*β* = −1.29, SE ± 0.35), and Reconyx camera traps (*β* = −7.56, SE ± 21.74) for detecting leopard (Figure 3 and File S1).

**TABLE 2:** Details of the model performed for factors influencing detection probability (*p*). We assumed occupancy (*ψ*) is constant. The top‐ranked models are shown as those with ΔAIC < 2.

Models	*K*	AIC	ΔAIC	AICwt	cumltvWt
*p* (CameraModel) *ψ* (.)	5	1155.08	0	0.72687	0.73
*p* (CameraModel + Occasion) *ψ* (.)	6	1157.04	1.96	0.27267	1
*p* (.) *ψ*(.)	2	1169.79	14.71	0.00047	1

*Note:* AIC = Akaike information criterion, AIC wt. = the AIC model weight, K = Number of model parameters including intercepts and covariates, ΔAIC is the difference in AIC values between each model and the model with the lowest AIC, *ψ* is the probability of occupancy, Camera Model = Types of camera model used in the survey, occasion = Sampling occasion.

### 2.2 Probability of Occupancy

We incorporated the detectability models into subsequent analyses to construct the occupancy model (Table 3). The best model for occupancy probability was “*p* (CameraModel) *ψ* (prey + settlement + river)” that included prey index (prey), distance to settlement (settlement), distance to river (river) in the BBC (ΔAIC = 0). The model‐averaged estimate of the habitat use probability of leopard in BBC to be 0.55 (SE ± 0.134, 95% CI: 0.234–0.773).

**TABLE 3:** Role of covariates on habitat use probability of leopard in Bardia—Banke Complex, on the basis of the constant detection probability *p* (CameraModel). The models are shown ranked by ΔAIC values.

Models	*K*	AIC	delta	AICwt	cumltvWt
*p* (CameraModel) *ψ* (settlement + river + prey)	9	1143.57	0	0.57272	0.57
*p* (CameraModel) *ψ* (road + river + prey)	9	1145.48	1.91	0.22084	0.79
*p* (CameraModel) *ψ* (river)	7	1146.31	2.73	0.1459	0.94
*p* (CameraModel) *ψ* (road)	7	1150.54	6.96	0.01761	0.96
*p* (CameraModel) *ψ* (settlement)	7	1151.1	7.53	0.0133	0.97
*p* (CameraModel) *ψ* (settlement + prey + Tiger)	9	1151.85	8.28	0.00914	0.98
*p* (CameraModel) *ψ* (NDVI)	7	1152.55	8.98	0.00643	0.99
*p* (CameraModel) *ψ* (NDVI + popden + Tiger)	9	1153.32	9.75	0.00438	0.99
*p* (CameraModel) *ψ* (TRI + settlement + waterholes)	9	1155	11.43	0.00189	0.99
*p* (CameraModel) *ψ* (Habitat)	8	1155.28	11.71	0.00164	0.99
*p* (CameraModel) *ψ* (popden)	7	1155.48	11.9	0.00149	1
*p* (CameraModel) *ψ* (Habitat + prey + Tiger)	10	1156.16	12.58	0.00106	1
*p* (CameraModel) *ψ* (Habitat + TRI + Tiger)	10	1156.64	13.07	0.00083	1
*p* (CameraModel) *ψ* (Tiger)	7	1156.88	13.31	0.00074	1
*p* (CameraModel) *ψ* (.)	6	1157.04	13.47	0.00068	1
*p* (CameraModel) *ψ* (prey)	7	1157.05	13.47	0.00068	1
*p* (CameraModel) *ψ* (waterholes)	7	1158.03	14.46	0.00041	1
*p* (CameraModel) *ψ* (TRI)	7	1159.04	15.47	0.00025	1

*Note:* AIC = Akaike information criterion, AICwt. = the AIC model weight, *K* = Number of model parameters including intercepts and covariates, ΔAIC = the difference in AIC values between each model, *ψ* is the probability of habitat use, settlement = Distance to the human settlement, CameraModel = Types of camera model used in the survey, road = Distance to road, river = Distance to river, Habitat = Types of habitat, TRI = Terrain Ruggedness Index, NDVI = Normalized Diversity Vegetation Index, Tiger = Occurrence of tiger in the grid, prey = Prey index, popden = Population density, waterholes = distance to waterholes (artificial ponds).

**FIGURE 4:**
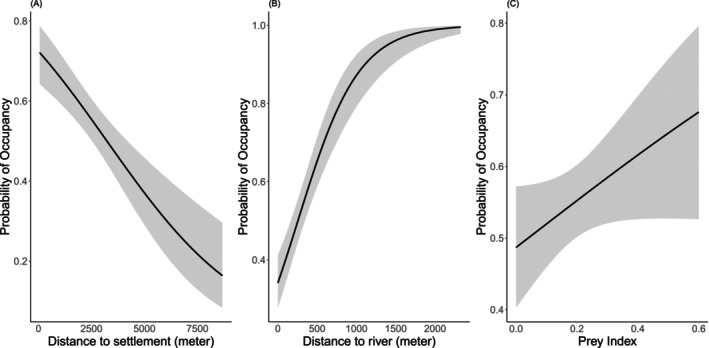
The probability of leopard habitat use in relation to the covariates: (A) Distance to settlement, (B) Distance to river, and (C) Prey Index.

The model–specific *β*‐coefficient values for prey 1.11 (SE ± 1.04), distance to settlement −2.68 (SE ± 1.15), and distance to river 6.03 (SE ± 3.70) (Figure 4 and File S2). The leopard habitat use was positively influenced by river and prey index, whereas it was negatively associated with distance to settlement.

**FIGURE 5:**
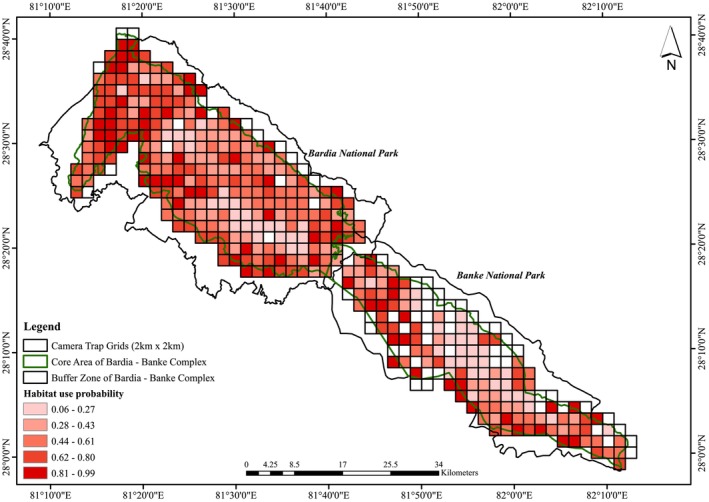
Leopard habitat use probability map of Bardia—Banke Complex. Color gradient from light to dark indicates low to high probability of leopard habitat use.

The spatial representation of leopard habitat use (Figure 5) indicates higher habitat use in the area with high prey density and the Karnali floodplain of BNP and the Rapti‐Gavar area of BaNP, following up with the nearby settlement area (edge of the park).

## 3 Discussion

Our study highlights the fine‐scale habitat use of leopard in the western TAL, representing within the home‐range habitat selection pattern derived from camera trap by identifying the covariates influencing occupancy and detection probability. Our findings shed light on the naïve occupancy of 0.20, and a model–averaged occupancy estimate of 0.55 (SE ± 0.134, 95% CI: 0.234–0.773), thus indicating that leopards were moderately distributed across the BBC. The estimated occupancy (*Ψ* = 0.55) in BBC is comparable to other tiger‐range areas and countries, where leopards share habitats with tigers. Joshi et al. (2025) reported leopard occupancy of 0.66 from Shuklaphanta National Park; Lamichhane et al. (2021), recorded 0.57 in Chure range of Nepal. Additionally, Katuwal et al. (2025) found low leopard occupancy (0.17 ± 0.04) in Parsa–Koshi Complex (PKC), with greater occupancy rates within protected areas of Nepal. Also, Thapa et al. (2021) concluded that across the broader TAL, leopard occupancy declined substantially from 0.86 in 2009 to 0.59 in 2013, with deforestation and road development negatively affecting both occupancy and detection.

### 3.1 Detection and Camera Trap Efficiency

Our findings show that camera model type strongly influenced detection probability, with Browning camera performing significantly better than Panthera, Cuddeback, and Reconyx. According to the variation in sensitivity, trigger speed, and detection range among the camera trap models, influence the detection rates (Burton et al. 2015; Meek et al. 2015). Contrasting to our result, from the same study area, Shah et al. 2025a, 2025b during the occupancy analysis of tiger concluded that Reconyx camera performed well for the detection of the tigers. This study accounts for camera‐specific detectability can introduce significant bias into occupancy and density estimates (Rovero and Kays 2021), also, camera model can be a more important covariate than many environmental variables (Sollmann et al. 2013). Also, the camera trap deployment duration per station was relatively short for a large carnivore's survey. Although this likely contributed to the low detection probabilities observed. Hamel et al. (2013) found that across multiple species, a threshold of 20–30 free days was required to stabilize occupancy and detection probability estimates. For leopards specifically, Harmsen, Saville and Foster (2021) showed that a 12‐year camera trap study revealed an abundant population that would have been incorrectly assessed as rare if studied short‐term, highlighting how brief surveys can underestimate populations because of low detection rates.

### 3.2 Ecological Drivers of Habitat Use

The positive association between leopard habitat use and prey availability is consistent with global evidence (Chaudhary et al. 2020; Gubbi et al. 2020; Searle et al. 2020). In Africa's Kavango‐Zambezi Transfrontier Conservation Area, leopard habitat use was positively influenced by prey availability, with relative abundance specifically associated with steenbok availability (Searle et al. 2020). Similarly, in India's Gir protected area, leopard site use intensity showed positive associations with multiple prey species including chital, nilgai and sambar (Chaudhary et al. 2020). Gubbi et al. (2020) suggested that leopard space‐use increased with proportion of natural habitats and the presence of large wild prey. Similarly, coinciding with our result, Katuwal et al. (2025) also recorded occupancy of leopard was positively associated with prey from Parsa‐Koshi Complex. Lamichhane et al. (2021) findings from Chure range aligned with our results stating that prey availability is positively influencing the leopard habitat use.

As hypothesized, leopard's habitat use was negatively influenced by the distance to human settlement, indicating higher use of areas closer to settlements (*β* = −2.68 SE ± 1.15). Furthermore, leopard's habitat use increased with higher population density (*β* = 7. SE ± 10.25). Similarly, leopard occupancy was higher near human settlements in Shuklaphanta National Park (Joshi et al. 2025). Additionally, in Bhutan, leopard‐dhole co‐occupancy probability increased in areas with higher human settlement density (Penjor et al. 2022). Leopard can persist in highly modified landscape with high human population density (Athreya et al. 2013, 2016; Kuhn 2014). Similar, positive association with human population density was observed by Lamichhane et al. (2021) in Chure, Nepal. The observed positive association should not be considered as true coexistence but might be an outcome of complex social‐ecological interactions between leopard, humans, in human‐modified landscape, particularly in light of the increasing incidence of human‐leopard conflict (Acharya et al. 2016). Rather, it likely reflects leopards' remarkable behavioral flexibility and relaxed avoidance of human activity in human‐modified landscapes (Pilfold et al. 2024). In the BBC, settlements are embedded within a mosaic of croplands, livestock sheds, and community forests that inadvertently offer cover and alternative prey, drawing leopards into human settlements (e.g., Poudel et al. 2023). Local livestock management practices such as free grazing, and poorly secured corals further increase the attractiveness to leopards. Records of livestock depredation from the fringe area of the BBC (Wegge et al. 2018; Kandel et al. 2020; Subedi et al. 2020; Poudel et al. 2023; Paudel et al. 2024; Chaudhary et al. 2025) further substantiate the fact that leopard use of human‐dominated areas is therefore a precarious form of overlap rather than coexistence between leopard and human with clear implications for targeted conflict‐prevention strategies in such shared landscape.

Our result highlighted a strong positive influence of distance to river on leopard habitat use, suggesting that leopards are more likely to occur in areas farther from riparian zones. This pattern is consistent with findings from the southern Kalahari, where Bothma and Le Riche (1984) highlighted that leopards are independent of surface water, indicating they can survive in diverse habitats. The avoidance of riparian zones in the BBC may therefore reflect not a need for water, rather leopard preference for the rugged, broken terrain that characterizes areas away from rivers, and supported by a positive relationship between leopard habitat use and TRI (*β* = 0.26 SE ± 1.10). Rugged terrain provides leopards with rocky outcrops and broken topography that serves as denning sites, ambush cover, and refugia from larger competitors (Joshi et al. 2025). Similar terrain‐driven habitat selection has been documented in Nepal's Chure range, where ruggedness was a key positive covariate of leopard occupancy, highlighting occupancy was higher in rugged terrain (Lamichhane et al. 2021). However, in the BBC, terrain complexity likely plays a secondary role relative to prey distribution and proximity to settlements.

Our findings contrast from the prevailing view among local communities and practitioners that dense tiger populations in core areas tend to push leopards toward the fringes. Contrary to our initial hypothesis, we recorded a positive but nonsignificant association of leopard occupancy with the tiger presence in BBC (*β* = 1.70 SE ± 1.66, CI: −1.55 to 4.95). While there is variation in this pattern between states of avoidance and coexistence (beta confidence interval overlaps zero), there is a tendency toward a mean coexistence response between leopards and tigers. This pattern is likely facilitated by the availability of prey (DNPWC and DFSC 2022), allowing these two large carnivores to coexist especially in the Karnali floodplain, where the prey density is high (File S1) despite potential interference competition. Evidence from African large carnivore systems further supports the possibility of spatial coexistence between dominant (Lions *Panthera leo* ) and subordinate predators (leopards), highlighting independent patterns of habitat use when prey availability and habitat complexity are sufficient to reduce direct competitive suppression (Miller et al. 2018). Although our study showed spatial overlap, temporal partitioning of activities may have facilitated their coexistence in the area. As tigers are socially dominant predators and influence temporal activity patterns of other co‐occurring carnivores (Karanth and Sunquist 2000; Odden et al. 2010; Harihar et al. 2011; Lamichhane et al. 2019). Comparable results were reported by Katuwal et al. (2025) in PKC, eastern Nepal, particularly in areas with high prey density. High prey biomass appears to be the primary factor enabling peaceful coexistence between tigers and leopards (Lovari et al. 2015), highlighting the importance of maintaining abundant prey populations for supporting sympatric large carnivores in the area. Odden et al. (2010) suggested that interference competition between leopard and tiger was the issue, but not competition for food was a limiting factor from the Karnali floodplain of BNP. In contrast, spatial segregation has been documented in Shuklaphanta National Park, where leopard detection probability was negatively correlated with tiger presence, where they exhibit greater tolerance to human disturbance and rugged terrain (Joshi et al. 2025). These findings indicate that tiger‐leopard interactions are context‐dependent, shaped by prey availability, habitat characteristics, and anthropogenic pressures. Understanding these factors is critical for effective management of sympatric large carnivores. These insights highlight the importance of maintaining prey‐rich habitats and heterogeneous landscapes to support the coexistence of sympatric large carnivores.

## 4 Conclusion

Our study demonstrates that leopard occupancy in the BBC was primarily shaped by prey availability and anthropogenic gradients, particularly distance to settlements and distance from rivers. These variables consistently emerged as the strongest predictors of habitat use at the 4 km^2^ grid scale. The absence of a strong negative association with tiger activity indicates that leopard occupancy in the BBC is more strongly influenced by bottom‐up processes (prey distribution) than by top‐down competitive exclusion at the spatial grain examined. The tendency to occur closer to settlements further suggests behavioral flexibility and adaptability to human‐modified environments. Leopards' ability to use areas near human settlements presents both a challenge and an opportunity for conservation. This underscores the urgent need for community‐based conflict mitigation strategies, such as livestock management, improved predator‐proof corals (as perceived effective against livestock depredation) (Shrestha et al. 2025), insurance schemes (Sherchan et al. 2022), and awareness programs (Kadariya et al. 2023). This research provides a valuable baseline for monitoring the population and evaluating the effectiveness of conservation interventions in the Bardia—Banke Complex and similar human‐felid interface landscapes across South Asia.

## Author Contributions

**Rabin Bahadur K. C.:** conceptualization (lead), data curation (equal), formal analysis (lead), investigation (equal), methodology (lead), writing – original draft (equal), writing – review and editing (lead). **Jyoti Sharma:** conceptualization (equal), data curation (lead), formal analysis (equal), investigation (lead), methodology (equal), writing – original draft (lead), writing – review and editing (equal). **Bed Kumar Dhakal:** methodology (equal), supervision (equal), writing – review and editing (equal). **Rabin Kadariya:** methodology (equal), supervision (equal), validation (equal), writing – review and editing (equal). **Ajit Tumbahangphe:** methodology (equal), writing – review and editing (equal). **Shyam Kumar Thapa:** conceptualization (equal), investigation (equal), methodology (equal), validation (equal), writing – original draft (equal), writing – review and editing (equal). **Naresh Subedi:** conceptualization (equal), funding acquisition (lead), methodology (equal), supervision (lead), validation (lead), writing – review and editing (equal).

## Ethics Statement

We did not carry out any experiments with live animals. Field surveys and data collection were conducted with prior approval from the DNPWC. We properly acknowledged supporting organizations for this research.

## Conflicts of Interest

The authors declare no conflicts of interest.

## Supporting information


**Appendix S1:** ece373285‐sup‐0001‐supinfo.zip.

## Data Availability

Data associated with this manuscript can be accessed at the Zenodo data repository (https://doi.org/10.5281/zenodo.17763483).
