# Supplementary material for: Ecological Factors at a Fine Spatial Scale Influencing Leopard (Panthera pardus) Habitat Use in the Bardia–Banke Complex, Nepal
Source: Ecol Evol. 2026 Mar 18;16(3):e73285. doi: 10.1002/ece3.73285 (PMC13093819; doi:10.1002/ece3.73285)
Supplement: Supplementary file 1 — Appendix S1: ece373285‐sup‐0001‐supinfo.zip. [file ECE3-16-e73285-s001.zip › Supplementary File 2.docx]

**Ecological Factors at a Fine Spatial Scale Influencing Leopard (*Panthera pardus*) Habitat Use in the Bardia – Banke Complex, Nepal**

Rabin Bahadur K.C.^1*^, Jyoti Sharma^2^, Bed Kumar Dhakal^3,4^, Rabin Kadariya^1^, Ajit Tumbahangphe^1^, Shyam Kumar Thapa^5^, Naresh Subedi^1*^

^1^National Trust for Nature Conservation, Lalitpur, Nepal

^2^University of Inland Norway, Norway

^3^Department of National Parks and Wildlife Conservation, Kathmandu, Nepal

^4^Tribhuvan University, Kathmandu, Nepal

^5^Zoological Society of London, Nepal Office

***Corresponding Author:** [rbn.kc80@yahoo.com](mailto:rbn.kc80@yahoo.com); [nareshsubedi@gmail.com](mailto:nareshsubedi@gmail.com)

**Supplementary File 2: The relative strength and direction of influence by the selected covariates on the leopard habitat use and detection.**

| **Category** | **Covariates** | **Beta** | **Error** | **LCI** | **UCI** |
| --- | --- | --- | --- | --- | --- |
| Occupancy | Settlement | -2.68 | 1.15 | -4.93 | -0.43 |
|  | River | 6.03 | 3.70 | -1.23 | 13.29 |
|  | Prey | 1.11 | 1.04 | -0.92 | 3.14 |
|  | Road | -2.46 | 1.24 | -4.89 | -0.04 |
|  | TRI | 0.26 | 1.10 | -1.89 | 2.42 |
|  | Waterholes | -0.54 | 1.47 | -3.42 | 2.34 |
|  | NDVI | -5.67 | 3.82 | -13.15 | 1.81 |
|  | Population density | 7.00 | 10.25 | -13.08 | 27.08 |
|  | Tiger | 1.70 | 1.66 | -1.55 | 4.95 |
|  | Habitat Flat | 0.70 | 0.53 | -0.34 | 1.74 |
|  | Habitat Hilly | -0.59 | 0.57 | -1.70 | 0.53 |
|  | Habitat Streambed | -1.66 | 0.73 | -3.09 | -0.23 |
|  | Camera Model Browning | -3.23 | 0.25 | -3.71 | -2.75 |
| Detection | Camera Model Cuddeback | -0.78 | 0.24 | -1.25 | -0.30 |
|  | Camera Model Panthera | -1.29 | 0.35 | -1.98 | -0.60 |
|  | Camera Model Reconyx | -7.56 | 21.74 | -50.17 | 35.05 |
|  | Occasion | 0.00 | 0.01 | -0.02 | 0.03 |
