# Supplementary figures and images for: Ecological Factors at a Fine Spatial Scale Influencing Leopard (Panthera pardus) Habitat Use in the Bardia–Banke Complex, Nepal
Source: Ecol Evol. 2026 Mar 18;16(3):e73285. doi: 10.1002/ece3.73285 (PMC13093819; doi:10.1002/ece3.73285)

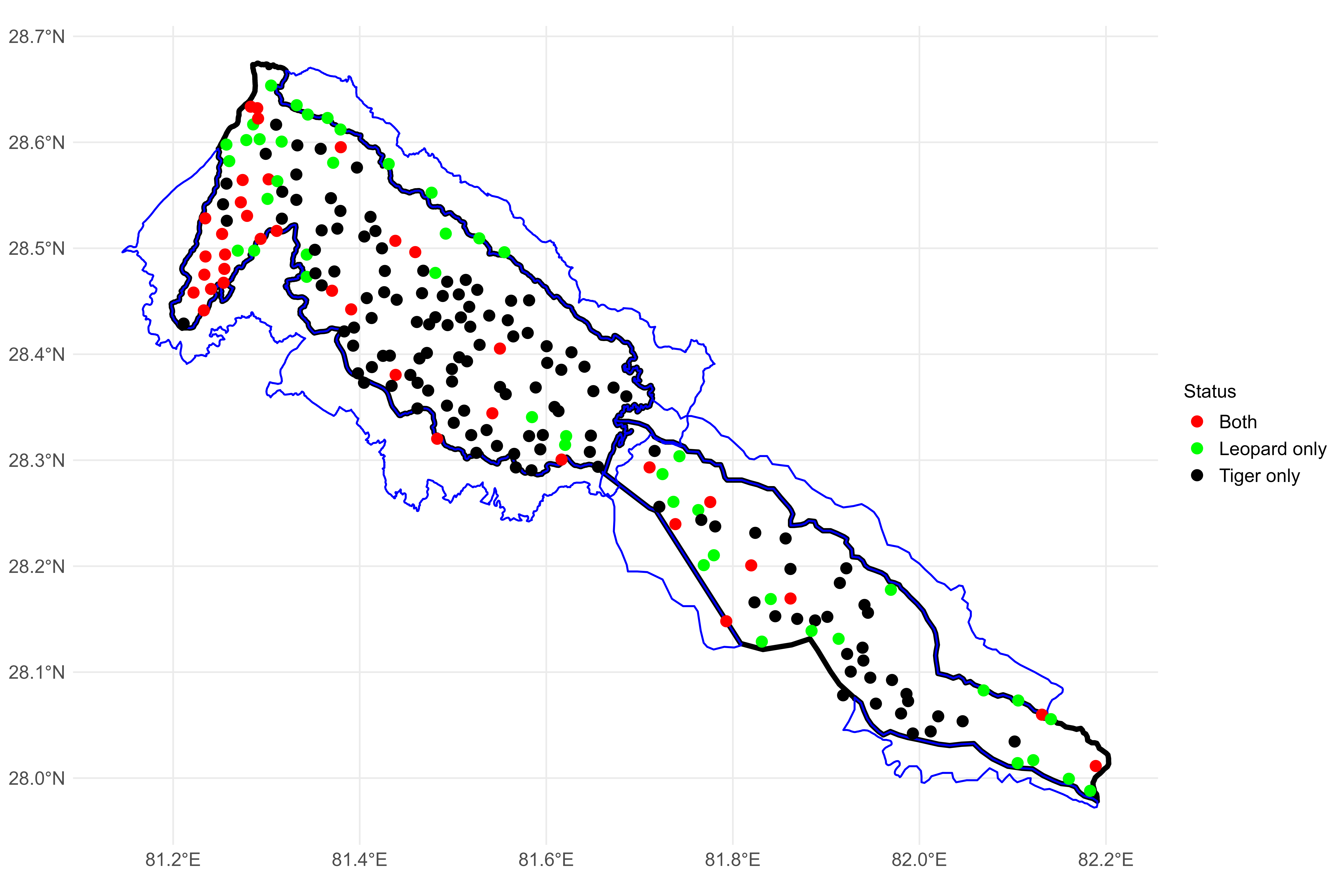

Supplement: Supplementary file 1 — Appendix S1: ece373285‐sup‐0001‐supinfo.zip. [file ECE3-16-e73285-s001.zip › Supplementary File 1.png]
